# The 24th Annual Meeting of the Rocky Mountain Virology Association

**DOI:** 10.3390/v17020262

**Published:** 2025-02-14

**Authors:** Kaitlyn R. Dirks, Samantha M. Pinto, Kylee N. Pham, Talia J. Byrne-Haber, Ryan W. Thompson, Oshani C. Ratnayake, Joel Rovnak, Rushika Perera

**Affiliations:** Department of Microbiology, Immunology, and Pathology, Colorado State University, Fort Collins, CO 80523, USA; kaitlyn.dirks@colostate.edu (K.R.D.); samantha.pinto@colostate.edu (S.M.P.); kylee.pham160@gmail.com (K.N.P.); talia.byrnehaber@colostate.edu (T.J.B.-H.); ryan.thompson@colostate.edu (R.W.T.); oshani.ratnayake@colostate.edu (O.C.R.); joel.rovnak@colostate.edu (J.R.)

**Keywords:** prion, intestinal homeostasis, arboviruses, vaccines

## Abstract

Located in the Rocky Mountains within the Arapahoe and Roosevelt National Forests, Colorado State University’s Mountain Campus in Pingree Park hosted the 24th Annual Rocky Mountain Virology Association’s meeting in 2024. A total of 165 participants, both regional and international, participated in the 3-day event, which consisted of 48 talks and 42 posters. These presentations discussed developments in prion research, current affairs, and novel tools in virology; investigated arboviruses and their vectors, as well as molecular foundations of viral interactions; and provided increased understanding of viral immunology and vaccines. This year’s Randall Jay Cohrs keynote presentation unveiled how viral infections disrupt intestinal homeostasis via Sting-dependent NK-kB signaling. This novel research demonstrated the importance of immunological pathways in the virus-induced disruption of homeostasis. Nested in the valley of the Rocky Mountains, participants could enjoy the fall colors and partake in hiking and fishing all while discussing science and networking amongst a variety of scientists. This report encapsulates selected presentations from the 24th Annual Rocky Mountain Virology Association meeting.

## 1. Introduction

**Figure 1:**
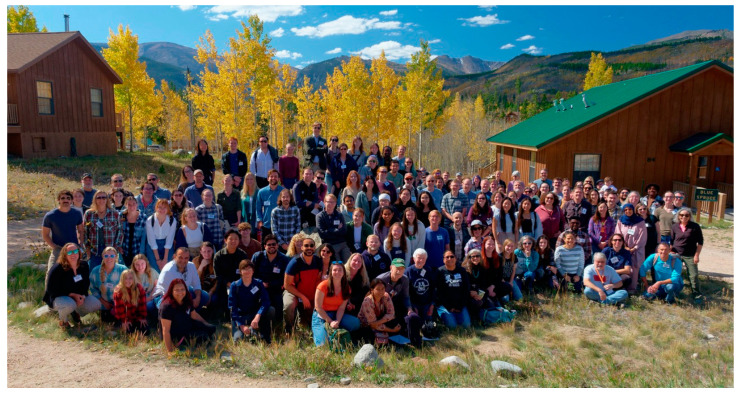
Attendees of the 24th Rocky Mountain Virology Association meeting.

With the goal of providing a platform for international and regional scientists to advance virology, the Rocky Mountain Virology Club (RMVC) was established in 2000. The club aimed to provide a forum for virologists, at any stage in their careers, to collaborate and share their work. Coinciding with developments in virology, the club expanded its doors and added prion biologists in 2009. A year later, in 2010, the RMVC was named the Rocky Mountain Virology Association (RMVA). Since the first meeting in 2001, there has been a steady growth in participants, with this year’s participation reaching 135 attendees pictured in Figure 1. Participants gathered at the colorful Colorado State University Mountain Campus from 27 September to 29 September 2024. The conference featured five sessions, seven invited speakers, 48 oral presentations, and 42 poster presentations.

The meeting began with Dr. Carla Saleh, from the Institute Pasteur in Paris, presenting the Randall Jay Cohrs keynote address on how viral infections disrupt intestinal homeostasis via Sting-dependent NK-kB signaling. This year’s national and international invited speakers included Dr. Szymon Manka for the Richard A. Bessen lecture on prion shape and spread, Dr. Ian Hogue’s presentation on alpha herpesvirus release, Dr. Molly Ohainle’s presentation on intracellular barriers to lentiviral infections, Dr. Daniel Blanco-Melo’s presentation on the multiomic characterization of past viral infections, and Dr. Kristian Andersen’s presentation on the COVID-19 pandemic’s origin.

Poster presentations were introduced through lightning talks. These talks include an informal presentation of less than 5 min accompanied by one PowerPoint slide, including any information from the scientific poster. Through poems, skits, and interpretive dances, these talks brought about many laughs and smiles. Like previous years, the RMVA showcased virology, prion biology, collaboration, mentorship, and fun. Selected abstracts are presented below.

## 2. Summary of Scientific Sessions

### 2.1. The Randall Jay Cohrs Lecture—Keynote Address

Dr. Carla Saleh from the Virus and RNA Interference in the Department of Virology at the Pasteur Institute presented her labs’ research on how viral infection disrupts intestinal homeostasis via Sting-dependent NK-kB signaling. The intestinal epithelium plays essential roles in defense against orally acquired pathogens. Homeostasis in this tissue is maintained by constant renewal of differentiated epithelial cells by basal levels of intestinal stem cell (ISC) proliferation and differentiation. She discussed how persistent viral infection reduces lifespan by driving intestinal dysfunction in a manner involving the sustained over-proliferation of ISCs. She showed that enteric viral infection accelerates aging by stimulating the prolonged activation of inflammatory Sting-Relish signaling, resulting in the dysregulation of classical epithelial repair systems and the loss of intestinal homeostasis. No animals or humans were used as subjects for this study. This work was supported by funding from the French Government’s Investissement d’Avenir program, Laboratoire d’Excellence Integrative Biology of Emerging Infectious Diseases (grant ANR-10-LABX-62-IBEID), the Agence Nationale de la Recherche (grant ANR-23-CE15-0038-01, INFINITESIMAL), Fondation iXcore-iXlife-iXblue Pour La Recherche, and DIM One Health (project no. R17043DJ—Allocation no. RPH17043DJA) to M.-C. Saleh. This project received funding from the European Union’s Horizon 2020 research and innovation program under the Marie Skłodowska-Curie grant agreement no. 101024099 to J.C.N.

### 2.2. Current Affairs and Novel Tools in Virology

Grace Ducharme, in tandem with Andrew Lingley, Rea Joshi, Rachel Conrad, Camden Long, Stephanie McCalla, and Emma Loveday of the Chemical and Biological Engineering Department of Montana State University, conducted this project aiming to simplify and execute on-chip, in-drop quantitative polymerase chain reaction (qPCR). The experimental method of qPCR enables researchers to amplify nucleic acids to measure gene expression over time. Current qPCR methods lack the capability to execute this technique within microfluidic drops, limiting our ability to evaluate heterogeneity in gene expression at the single-cell level. Combining previous research from the Loveday and McCalla labs, their goal was to design a microfluidic device enabling qPCR for single-cell gene expression analysis. The main challenge of this project was addressing the instability of aqueous microfluidic drops during temperature fluctuations necessary for qPCR. To combat this problem, the devices were constructed with various materials, including 3D-printed resin, glass, and SU-8 as opposed to the traditional microfluidic device polymer, PDMS, which tends to destabilize at high temperatures. This project involved utilizing computer-aided design (CAD) platforms to design various device geometries and carry out the subsequent fabrication of the devices and evaluation of their overall performance. The successful development of these devices will enable the quantification of nucleic acids from single cells, facilitating the monitoring of heterogeneous cell lines. Through interdisciplinary collaboration and by utilizing previous research in drop-based microfluidics and single-cell qPCR, the objective of this project was to develop a system capable of performing on-chip single-cell qPCR reactions. No animals or humans were used as subjects for this study. Funding for this project stemmed from the MSU USP Scholarship, NNCI member supported by NSF Grant ECCS-2025391, the USA National Institutes of Health and the National Institute of Allergy and Infectious Diseases (NIH R21 AI178432-01), and the Center of Excellence in Influenza Research and Surveillance contract from Johns Hopkins University (HHS N7593021C00045).

Treana Mayer, working alongside Jourdan Ringenberg, Krista Dilione, Julianna Lenoch, Sue VandeWoude, and Sarah Bevins in the Department of Microbiology, Immunology, and Pathology of Colorado State University, conducted a study researching H5N1 (2.3.4.4b). Highly Pathogenic Avian Influenza has been devastating to the poultry industry and wild bird populations in the USA since 2021, with highly concerning mammalian spillover and cattle transmission. Domestic and wild cats have experienced fatal neurologic disease following H5N1 infection, but their relative importance as bridging hosts for domestic–wildlife–human transmission is unknown. They summarized the descriptive spatial associations of confirmed USA H5N1 cases in felids in relation to wild bird or agricultural outbreaks to begin questioning the landscape epidemiology of feline spillover. County-level data from 76 domestic and wild felids with H5N1, >73 k wild birds screened under active and passive surveillance for Influenza A, and >100 M poultry affected by outbreaks were included from December 2021 to April 2024. All samples were screened via Influenza A PCR, subtyped, and sequenced for confirmation by the National Veterinary Services Laboratory. The pooled county-level prevalence in wild birds was compared using H5 detections to all screening tests, with county sums of affected domestic birds. Data visualization and hot spot analysis performed in ArcGIS detected statistically significant hot and cold spots of the virus from wild and domestic avian sources. Feline cases were summarized by number of cases, species, and whether there was a known agricultural link. Spatially, 29 (38%) felid cases occurred in counties with no associated avian detections, with 12 domestic cat clusters being associated with agricultural sources. Hot spots of avian sources of the virus, wild or domestic, did not explain the spatial patterns of feline infections, even for wild felids. In contrast, only recently (Spring 2024) have increased agricultural-associated infections been described. While these results may represent missing data, additional environmental or species transmission risks, and/or changing viral dynamics, felids may be useful sentinels in under-surveilled counties. This complex disease issue with rapid evolution and spread requires continued monitoring to understand mammalian spillover and pandemic preparedness. Neither humans nor animals were used as research subjects for this study. Funding was provided by the USDA NWRC cooperative agreement (AP23WSNWRC00C107).

### 2.3. Developments in Prion Research

Jesse Cole, along with Erin McNulty, Amy V. Nalls, Joseph Westrich, and Candace K. Mathiason (Department of Microbiology, Immunology, and Pathology, Colorado State University), explained their work studying the relationship between extracellular vesicles (EVs) and the mechanisms of Chronic Wasting Disease (CWD) peripheralization. CWD is the most efficiently transmitted prion disease and is currently detected in captive and free-ranging cervid populations in 35 states in the USA, five Canadian Provinces, Europe, and Asia. The infectious agent that causes disease has been detected in the blood of prion-infected hosts. As CWD prions traffic across mucosal surfaces into the blood stream within minutes post oral consumption, there is an increased need to determine the role blood-borne prions play in disease initiation and pathogenesis. The research group aimed to elucidate the relationship between extracellular vesicles (EVs) and the mechanisms of CWD peripheralization. EVs are nanoparticles known to be released from virtually all cell types and have been demonstrated to facilitate intercellular communication via the transport of nucleic acids, lipids, and proteins between cells. They hypothesized that EVs facilitate the hematogenous dissemination of prions, contributing to the dispersal of the infectious agent to tissues with the capacity to support disease initiation and progression. Differential centrifugation, size exclusion chromatography (SEC), immunoblotting, transmission electron microscopy (TEM), bicinchoninic acid assay (BCA), and nanoparticle tracking analysis (NTA) were performed to isolate, characterize, and quantify blood serum-derived EVs. Real-time quaking-induced conversion (RT-QuIC) and serial protein misfolding cyclic amplification (sPMCA) were performed to assess the presence of prion seeding activity (prions) in EV samples. Blood serum-derived EVs were harvested from naïve and CWD-infected white-tailed deer. An analysis of the presence of amyloid seeding (prions) within EV samples is ongoing. This, to their knowledge, demonstrates the first characterization of EVs in a cervid model. These studies will provide the basis for the investigation of CWD peripheralization in the host. This work was funded by the NIH Medical Scientist Training Program (MSTP) T32 Fellowship (5T32GM136628-04), NIH–NIAD 2R01AI112956-06, 1R01AI156037, and 2P01AI077774. No animal or human studies were performed.

Benjamin S. Steadman ^1^, with Jifeng Bian ^2^, Ronald A. Shikiya ^1^, and Jason C. Bartz ^1,3^ (^1^ Department of Medical Microbiology and Immunology, School of Medicine, Creighton University; ^2^ Virus and Prion Research Unit, National Animal Disease Center, United Stated Department of Agriculture—Agricultural Research Services; ^3^ Department of Microbiology, Immunology, and Pathology, Prion Research Center, Colorado State University), presented his research on minor prion strains and their conversion efficiency compared to other strains. Infectious self-propagating prions result in fatal neurodegenerative diseases. Prion strains differ in heritable biochemical and pathological properties that can result in zoonotic transmission. Prions are dynamic quasispecies consisting of a dominant strain and minor strains. Recently, they directly observed pre-existing minor prion strains by selectively reducing PrPSc from hamster-adapted drowsy (DY) transmissible mink encephalopathy (TME). They found that minor prion strains differed from their parental strain, DY TME, in electrophoretic migration, antibody affinity, conformational stability, protease resistance, incubation periods, and clinical signs. Here, using protein misfolding cyclic amplification (PMCA), they investigated the intraspecies and interspecies conversion efficiency of minor prion strains compared to DY TME. They discovered that minor prion strains had a higher intraspecies PMCA conversion efficiency than their parental strain, DY TME. Similarly, minor strains more efficiently infected RK13 cells expressing hamster PrPC than DY TME. Although DY TME did not amplify after one to four serial rounds of PMCA, minor prion strains reliably converted mouse PrPC into PrPSc after a single round of PMCA. Together, these novel data provide evidence that minor prion strains can traverse transmission barriers. These findings also suggest that prior studies reporting unsuccessful attempts to traverse species barriers may be only investigating the transmission potential of the dominant strain without considering the capacity of minor prion strains to result in interspecies transmission. Furthermore, minor substrains differed from one another in intraspecies and interspecies PMCA conversion efficiency and cell conversion efficiency, suggesting that the strain diversity of prion quasispecies is greater than previously considered. This work was funded by the NIH NINDS R01NS133050 and the NIH NINDS R01NS103763. All animal studies were performed following the guidelines and protocols approved by the Institutional Animal Care and Use Committee of Creighton University.

SJ Stein ^1^, along with Yuan Qi ^1^, Glenn Telling ^2^, and Jason C. Bartz ^1^ (^1^ Department of Medical Microbiology and Immunology, Creighton University School of Medicine; ^2^ Prion Research Center, Department of Microbiology, Immunology, and Pathology, Colorado State University), shared their research on soil-bound prions. Chronic Wasting Disease (CWD) is a prion disease of cervids that effects the central nervous system and results in death. CWD is highly contagious, and the risk of zoonosis jeopardizes multiple sympatric mammalian species, including humans. CWD can bind to soil minerals as a reservoir, allowing them to persist and remain infectious for extended periods of time. Assessing the effect of soil-bound prions on interspecies transmission will answer fundamental questions about prion structure and transmission, ultimately aiding in the assessment of prion zoonotic potential. Montmorillonite and Kaolinite clay types were sorbed to CWD-positive and -negative Elk brain homogenate, and density-based centrifugation was performed using a sucrose cushion to separate prion–soil complexes and potentially unbound prions. Protein Misfolding Cyclic Amplification (PMCA) was used to analyze samples and determine the replicability of soil-bound prions and the relevance of unbound prions. Additionally, PMCA was used to explore the transmission efficiency to sympatric species. Clays were found to effectively sorb CWD-positive prions in 24 h. Soil-bound prions retained the ability to effectively replicate and were detected at higher limits than the controls, suggesting greater amounts of prion protein present in the prion–soil substrate. No unbound prions were detected in the soil–prion substrates, indicating that sorption capacity was not exceeded. Unbound prions in a soil-free control were unable to pellet through the sucrose cushion, demonstrating the effectiveness of this methodology. The described methodology can be utilized for the successful separation of soil-bound prions from unbound prions. The soil-bound prions replicated to higher detection limits than the unbound prions, indicating a potential structural change during the binding process. This research was funded by the NIH National Institute of Allergy and Infectious Diseases. All animal studies were performed following the guidelines and protocols approved by the Institutional Animal Care and Use Committee of Creighton University.

M. L. Tyer ^1^, with Juliana Sun ^1^, Sehun Kim ^1^, Xutong Shi ^1^, Sylvie Benestad ^2^, and Glenn Telling ^1^ (^1^ Department of Microbiology, Immunology, and Pathology, Colorado State University; ^2^ Norwegian Veterinary Institute), discussed their studies on the unusual biochemical characteristics of CWD in K109Q moose. The first case of Chronic Wasting Disease (CWD) in Europe was identified in Norway in 2016. In collaboration with the Norwegian Veterinary Institute, Tyer et al. identified the etiology of European CWD as being independent from North American CWD. This represents an exciting opportunity to study naturally emerging strains of prion disease. Unique among Norwegian CWD cases are those identified in moose possessing a lysine-to-glutamine polymorphism at codon 109 (K109Q). Unlike cases of wild-type moose CWD in Norway, CWD cases in K109Q moose display unusual biochemical characteristics. Notably, isolates from these K109Q moose poorly infected their existing gene-targeted mouse model, where the endogenous mouse PrP was replaced with cervid PrP (GtQ mice). Since isolates from wild--type Norwegian moose CWD can infect GtQ mice, this polymorphism likely affects the transmissibility of this abnormal strain of CWD. To model the pathogenesis of K109Q-CWD, they designed a new knock-in mouse model that replaces the endogenous mouse PrP sequence with the moose PrP sequence containing the 109Q codon. Supplementing this investigation as they established their K109Q mouse colony, they interrogated the susceptibility of the K109Q polymorphism in a cell-based infection assay. The results of these studies will help shed light on the pathogenesis and biochemical characteristics of emergent CWD strains. This work was funded by T32GM144856, 1R01NS121682, 1R01NS109376, and PO1-0011877A grants. All animal studies were performed following the guidelines and protocols approved by the Institutional Animal Care and Use Committee of Colorado State University.

### 2.4. Investigating Arboviruses and Their Vectors

Rachel E. Lange ^1,2^, Melissa A. Prusinski ^3^, Alan P. Dupuis II ^2^, and Alexander T. Ciota ^1,2^ (^1^ State University of New York University at Albany School of Public Health, Department of Biomedical Sciences, Albany, NY, USA; ^2^ The Arbovirus Laboratories, Wadsworth Center, New York State Department of Health, Slingerlands, NY, USA; ^3^ New York State Department of Health, Bureau of Communicable Disease Control, Vector Ecology Laboratory, Albany, NY, USA) performed research on the host-specific adaptation of Powassan virus to *Amblyomma americanum* and the role of PrM in tick-specific viral fitness. Powassan virus (POWV, family *Flaviviridae*) is a reemerging tickborne virus endemic in North America and Russia. POWV was first isolated in 1958 from a fatal encephalitic case in Canada. In 1997, a genetically distinct POWV-like agent was isolated from *Ixodes scapularis*. This revealed the existence of two lineages: lineage 1, Powassan virus (POWV-1), and lineage 2, deer tick virus (DTV). Each lineage is maintained in separate enzootic cycles, with POWV-1 thought to be primarily maintained between *I. cookei* and woodchucks and *I. marxi* and squirrels, while DTV is maintained between *I. scapularis* and small mammals. POWV-1/DTV, however, has been detected in a range of tick genera. In New York State (NYS), between 2018 and 2022, POWV-1 was isolated for the first time from *I. scapularis* and detected in *Dermacentor variabilis*, and DTV was isolated from *Amblyomma americanum*. The propensity for POWV-1/DTV to further adapt to new tick hosts is unknown but can facilitate the emergence of increasingly virulent strains. To understand the host-specific viral fitness of DTV in novel tick hosts, they conducted genetic and phenotypic characterizations of an *Amblyomma*-derived DTV strain from NYS. The genetic results show that this strain, DTV NY22-2958, contains a unique substitution in the premembrane (PrM) protein (L268F) and displays a clear fitness advantage in experimentally infected *A. americanum* nymphs. The growth kinetics in *A. americanum* cultures reveal an increased viral burst size associated with DTV NY22-2958. These data reveal the potential for POWV adaptation to a range of unique tick genera and suggest a role for PrM in species-specific adaptation. This research was funded with F31AI176725. No animals or humans were used when conducting this research.

Olivia M. Martinez, Emma K. Harris, Shelby Cagle, and Rebekah Kading, from the Dept. of Microbiology, Immunology, and Pathology, Colorado State University, conducted a study investigating the effects of temperature change on oviposition and the progeny viability of *Aedes aegypti* and *Culex tarsalis* mosquitoes. Temperature change is known to affect the transmission efficiency of mosquito-borne viruses, particularly those spread by *Aedes aegypti* and *Culex tarsalis* mosquitoes. Investigating how temperature impacts *Ae. aegypti* and *Cx. tarsalis* can inform future vector control and disease mitigation efforts, especially in the context of climate change. Their previous work showed impaired oviposition when Rift-Valley-fever-virus-infected adult *Ae. aegypti* mosquitoes were exposed to temperatures varying from typical environmental conditions. It is unclear whether this phenotype is caused by viral infection or abnormal temperatures. They hypothesized that temperature change negatively impacts survivorship, oviposition, and offspring development in uninfected *Ae. aegypti* and *Cx. tarsalis* mosquitoes. Blood-fed female mosquitoes (n = 50) were housed individually at lowered (18 °C), standard (28 °C), or elevated (32 °C) rearing temperatures. Oviposition was assessed by quantifying deposited eggs in comparison to withheld eggs obtained by ovarian dissection. Deposited egg hatch rates determine offspring viability. Preliminary data show increased oviposition and offspring developmental rates in *Ae. aegypti* at 32 °C compared to 28 °C. In contrast, *Cx. tarsalis* mosquitoes showed impaired egg deposition and developmental rates at 32 °C. Both species retained most of their eggs at 18 °C. Understanding the relationship between mosquito fecundity and temperature is important for anticipating vector disease dynamics in a complex global environment. This study was funded by the National Institute of General Medical Sciences of the NIH T34GM140958. No animal or human studies were performed.

Audrey Walker ^1^, Airn Hartwig ^2^, Jeffrey Marano ^2^, Angela Bosco-Lauth ^2^, and Richard Bowen ^2^ (^1^ Colorado State University, Department of Microbiology, Immunology and Pathology; ^2^ Colorado State University, Department of Biomedical Sciences) executed a study on the susceptibility and transmission potential of ectotherms and house sparrows to Japanese encephalitis virus (JEV). Japanese encephalitis virus (JEV) is a vector-borne flavivirus that is maintained in an enzootic lifecycle between mosquitoes, pigs, and wading birds. An estimated 68,000 human cases are reported annually, and symptoms in humans can range from a mild fever to severe neurological complications. Arboviral diseases are spreading to new areas at alarming rates secondary to increases in global trade and travel, climate change, and the migration of both animal reservoirs and vectors. Although JEV is currently only endemic in Asia, concerns for the spread of JEV to new areas are rising, as indicated by a recent outbreak on the mainland of Australia. This outbreak is causing significant public health impacts by inflicting illness in humans and creating notable economic losses to the pig industry. Given that other closely related arboviruses, such as West Nile virus, have spread to the USA over the past several decades, JEV holds high potential to become established in the USA However, little is known about what animal reservoirs, particularly wildlife inhabiting mosquito-dense locales, can contribute to JEV ecology in the USA. Here, they reported that ball pythons, garter snakes, and house sparrows are susceptible to JEV genotypes I and III, but not to JEV genotypes II and IV. However, frogs, toads, alligators, and green anoles are not susceptible to any of the four genotypes. Their results expand upon the knowledge base of susceptible species and provide evidence that domestic wildlife species can play a role in the introduction or maintenance of JEV within the USA. This research was funded by the USDA. All animal studies were performed following the guidelines and protocols approved by the Institutional Animal Care and Use Committee of Colorado State University.

### 2.5. Molecular Foundations of Viral Infections

Matthew J. Abbott, along with Heini M. Miettinen and Alyssa B. Evans from Montana State University’s Department of Microbiology and Cell Biology, presented his progress in evaluating viral reassortment in which orthobunyavirus genome segments are involved in neurovirulence. The California serogroup (CSG) of orthobunyaviruses is a group of genetically related tri-segmented RNA viruses, seven of which are encephalitic in humans, including La Crosse virus (LACV) and Inkoo virus (INKV). LACV is the leading cause of pediatric arboviral encephalitis in the USA and causes ~100 neuroinvasive disease cases annually. INKV, however, has only caused several confirmed neuroinvasive disease cases. Similarly, in murine models, LACV is highly neurovirulent, and INKV is mildly neurovirulent. The viral factors that mediate these disparate neurovirulence phenotypes are largely unknown. He created reassortant viruses between the L, M, and S segments of LACV and INKV to determine the genome segments involved in neurovirulence. He evaluated the replication kinetics and cytotoxicity of reassortant viruses in a human neuronal cell line in vitro and evaluated neurological disease in vivo in mice. Additionally, viral loads within the brain were analyzed during a time course infection. The in vivo results suggest that the LACV L segment, encoding the viral RNA-dependent RNA polymerase, and the M segment, encoding two envelope glycoproteins and a nonstructural protein, are both key mediators of neurovirulence, but neither drive neurovirulence alone. Replication kinetics assays showed some differences in growth kinetics correlated to the L and M segments; however, all viruses replicated to similar end titers. The results of cytotoxicity assays closely followed the in vivo results and suggest that the LACV M segment was associated with the ability to kill neurons. Together, their results indicate that interactions between the LACV L and M segments are the primary drivers of orthobunyavirus neurovirulence. This study was funded by Montana State University startup funds. All animal studies were performed following the guidelines and protocols approved by the Institutional Animal Care and Use Committee of Montana State University.

Annabel Anyang and Tyler Starr from the Department of Biochemistry, University of Utah, presented their work on the molecular evolution of zoonotic coronaviruses through intermediate hosts. Annabel explained that zoonotic spillover poses significant threats to global health, as seen in the MERS-CoV epidemic and the SARS-CoV-2 pandemic. Annabel further emphasized that understanding the zoonotic role of intermediate hosts, such as farmed minks and trafficked pangolins, is crucial, as these animals act as a bridge between wildlife and humans. Intermediate hosts can either amplify viruses in close human proximity, increasing the transmission chain to reach epidemic spread, or serve as evolutionary intermediates, facilitating viral adaptation from natural wildlife receptors to human orthologs. Both host scenarios increase the likelihood of zoonotic spillover. Their study aimed to elucidate the evolutionary mechanisms by which coronaviruses adapt through these intermediate hosts to enhance spillover potential. The authors specifically sought to identify the range of SARS-related and MERS-related coronaviruses capable of binding to, or evolving to bind, key receptors in minks and pangolins—two species known to harbor both viruses at the human–animal interface. In addition, they also sought to identify the mutational pathways that enable and enhance binding, assessing whether these adaptations correlate with the acquisition of human receptor tropism. Their approach includes a yeast surface display platform for high-throughput measurements of spike receptor affinities to understand the range of SARS-related and MERS-related coronaviruses naturally binding relevant mink and pangolin receptors. Furthermore, this group used deep mutational scanning libraries across phylogenetically dispersed coronaviruses to map the evolutionary landscape for receptor binding acquisition, revealing how these pathways vary across viral clades. Pseudoviral entry assays will validate the impacts of their novel mutations on viral entry via mink, pangolin, and human receptors. Structural modeling will further elucidate the biophysical changes these mutations induce, providing a comprehensive understanding of their role in viral adaptation. This work will highlight how human activities across the human–animal interface, like fur farming and wildlife trade, create epidemiological, ecological, and evolutionary conditions to drive spillover hotspots, with significant implications for predicting and mitigating zoonotic risks. This study was funded via the Searle Scholars Program. No animal studies were performed.

Talia Byrne-Haber, along with Phillida A. Charley, Laura Pulscher, Christie Mayo, Gilbert John, and Tony Schountz, from the Dept. of Microbiology, Immunology, and Pathology, Colorado State University, presented her research on animal and rodent surveillance of SARS-CoV-2 on Navajo Nation lands. Since the emergence of severe acute respiratory syndrome coronavirus 2 (SARS-CoV-2) in 2019, virus surveillance in humans and animals has become critical in understanding virus prevalence. Previously, the majority of animal virus surveillance occurred in urban areas. As such, less is known about the prevalence of these viruses in rural areas with free-range animals. The Navajo Nation supports a variety of wildlife and livestock that closely interact with the people living there. However, no system exists to surveil this area for animal diseases such as coronaviruses. Therefore, their study aimed to identify any coronaviruses prevalent in the livestock, companion animals, and wildlife living in rural areas on Navajo Nation land. To assess the presence of SARS-CoV-2, nasal, oral, and rectal swabs and sera from animals present on Navajo Nation land were collected. Additionally, tissue samples from a subset of deer mice in this area were collected. qRT-PCR, conventional RT-PCR, and sequencing targeting the SARS-CoV-2 Envelope (E) gene were used to determine prevalence. An ELISA was used to assess the sera for SARS-CoV-2 antibodies. Several animals and one deer mouse were positive for the E gene but negative in other areas of the genome. This led them to use pan-coronavirus primers, which are suggestive of coronavirus infection. All deer mice were seronegative for SARS-CoV-2, but one was positive for Sin Nombre virus. The characterization of positive samples is ongoing. This study provides an important baseline for coronavirus surveillance from the perspective of the animal–human interface on Navajo Nation land. This research was funded by the Animal and Plant Health Inspection Service (APHIS), project award no. AP23OA000000C020, from the U.S. Department of Agriculture’s Animal and Plant Health Inspection Service. All animal studies were performed following the guidelines and protocols approved by the Institutional Animal Care and Use Committee of Colorado State University.

Elizabeth A. Fortunato ^1^, along with Man I. Kuan ^1^, Lisa B. Caruso ^2^, John M. O’Dowd ^1^, and Italo Tempera ^2^ (^1^ Dept. of Biological Sciences, University of Idaho and ^2^ The Wistar Institute), presented her research on human cytomegalovirus. Human cytomegalovirus encodes over 100 genes. It has been known for years that the tegument protein pp71 disables the innate antiviral response of proteins DAXX and PML, allowing for immediate early viral gene expression. They previously reported that pp71 alone can induce site-specific DNA breaks, which occur as quickly as 15 min post infection. An analysis of the regions surrounding these break sites uncovered that pp71 directly bound to the cellular DNA and influenced the transcription of two cellular genes important in nervous system development, *NID1* and *MPZ*. To further define the spectrum of pp71-influenced cellular genes, she performed ChIP-Seq experiments in infected fibroblasts. Not unlike the herpesvirus regulatory proteins EBV EBNA1 and KSHV LANA, pp71 bound to a broad spectrum of cellular genes, approximately 300 in total. These genes cover a wide range of cellular functions. Several of these regulated genes were found to be cell type-specific, and their regulation requires full infection compared to the sole expression of pp71. The reach of pp71 is much broader than they had anticipated. The recent solution of the crystal structure of pp71 should assist in ascertaining important gene regulation residues within the DNA interface. *NID1* and *MPZ* are regulated even by an extremely low level of expression of pp71 in fibroblasts and Schwann cells, respectively, which is not true for other genes. Their focus was on these developmentally important genes, with the aim of thwarting pp71’s interaction with the DNA or rescuing these genes’ downregulation by their overexpression in the developing nervous system. This research was funded by the NIH R01AI139503. No animal or human studies were performed.

Geer C. ^1,2^, with Kapuscinski M. ^2^, Bankes A. ^2^, and Stenglein M. ^1,2^ (^1^ Cell and Molecular Biology Program, and ^2^ Dept. Of Microbiology, Immunology, and Pathology, Colorado State University), discussed her research on using protein structures to better understand virus reassortment in orthobunyaviruses. Viral reassortment occurs when two different segmented viruses co-infect a cell, and the viral genomes are mixed to produce a novel virus. Reassortment drives the evolution of viruses and is a major source of new and emerging human pathogens. The orthobunyavirus genus contains viruses with a tri-segmented single-stranded RNA genome that encodes a nucleoprotein (N protein) and viral polymerase (L protein). Previous research has shown that mismatched L and N proteins cannot function together. This presents a barrier to reassortment, but the molecular basis for this incompatibility is unknown. To better understand what sequences maintain L and N protein compatibility, she used a minigenome assay to test the function of La Crosse virus L protein with mismatched N proteins of viruses with varying percentages of sequence identity. They found that viruses with seventy percent or greater of sequence identity had some level of functionality between the L and N proteins, while sequences with lesser identity showed no protein activity. This experiment may help to define viral protein sequences that may be necessary for L-N interaction. In future experiments, she will mutate N sequences and create protein chimeras to functionally test candidate sequences. This research was supported by the NIH R01 AI177711. No animal or human studies were performed.

Adam Hafner ^1^ from the ^1^ Department of Microbiology and Immunology, University of Michigan Medical School, Ann Arbor, MI, USA, presented his work titled “Norovirus alters host metabolism for efficient virus replication” together with Noah Meurs ^2^, Ari Garner ^3^, Aditya Kannan ^1^, Harrison Wong ^4^, Li Zhang ^4^, Austin Wright ^5^, Costas Lyssiotis ^4^, Timothy J. Nice ^5^, Deepak Nagrath ^2^, and Christiane E. Wobus ^1^ (^2^ Department of Biomedical Engineering, University of Michigan, Ann Arbor, MI, USA; ^3^ Department of Microbiology, Immunology, and Inflammation, University of Illinois, Chicago, IL, USA; ^4^ Department of Cancer Biology, University of Michigan Medical School, Ann Arbor, MI, USA; ^5^ Department of Molecular Microbiology and Immunology, Oregon State University School of Medicine, Corvallis, OR, USA). In Adam’s talk, he emphasized that human noroviruses (HNoVs) are single-stranded positive-sense RNA viruses that are the leading cause of acute non-bacterial gastroenteritis. Despite the devastating public health impact of HNoV infections, neither vaccines nor antivirals exist, demonstrating the need for further investigations to better understand norovirus biology. Adam further explained how viruses hijack host metabolic pathways, allowing them to engineer more favorable intracellular environments to ensure optimal virion production. Additionally, the dysregulation of host metabolism during viral infections can significantly affect pathogenesis. However, little is known about the ability of noroviruses to reprogram host metabolic pathways. The recent metabolomics and quantitative flux analysis performed by the authors revealed that murine norovirus 1 (MNV-1) upregulates and relies on glycolysis and glutaminolysis for efficient virus infection in macrophages. The importance of these two pathways for optimal viral infection extends to numerous MNV strains and was validated in non-transformed macrophages and multiple cell types. Furthermore, follow-up investigations uncovered that genome replication is the stage of the MNV lifecycle most dependent on glutaminolysis. Early mechanistic studies have demonstrated that the activity of glutaminase, the rate-limiting enzyme in the glutamine catabolic pathway, is upregulated during MNV infections and that the overexpression of MNV NS1/2, but not the other nonstructural proteins, can mediate these changes. Current studies aim to extend these studies to HNoV and intestinal enteroids to uncover whether the observed metabolic alterations are virus- and/or cell-type-specific. Collectively, these data demonstrate the importance of host metabolic pathways for productive norovirus infection and the ability of a noroviral viral protein to alter the activity of a metabolic enzyme. This study was funded by the Molecular Mechanisms of Microbial Pathogenesis T32 Training Grant (5T32AI007528). No animal studies were performed.

Pathogenic hantaviruses are maintained world-wide within wild, asymptomatic rodent reservoir hosts, with increasingly frequent human spillover infections resulting in severe hemorrhagic fever disease. Despite limited experimental data, several hypotheses have arisen to explain limited or absent disease pathology in reservoir hosts. A study titled “Seoul orthohantavirus evades innate immune activation by reservoir endothelial cells” conducted by Alison M Kell, together with Stefan Klimaj, Autumn LaPointe, Kimberly Martinez, and Eduardo Hernandez Acosta at the Department of Molecular Genetics and Microbiology, University of New Mexico School of Medicine, Albuquerque, NM, USA, directly tested two leading hypotheses: (1) reservoir host cells induce a generally muted response to viral insults, and (2) these viruses employ host-specific mechanisms of innate antiviral antagonism to limit immune activation in reservoir cells. The researchers demonstrated that, in contrast to human endothelial cells, which mount robust antiviral and inflammatory responses to pathogenic hantaviruses, primary Norway rat endothelial cells do not induce antiviral gene expression in response to infection with their endemic hantavirus, Seoul orthohantavirus (SEOV). Reservoir rat cells, however, induce strong innate immune responses to exogenous stimulatory RNAs, type I interferon, and infection with Hantaan virus, a closely related hantavirus for which the rat is not a natural reservoir. Additionally, the authors found that SEOV-infected rat endothelial cells remain competent for immune activation induced by exogenous stimuli or subsequent viral infection. These findings support an alternative model for asymptomatic persistence within hantavirus reservoir hosts in which efficient viral replication within reservoir host cells prevents the exposure of critical motifs for cellular antiviral recognition and thus limits immune activation that would otherwise result in viral clearance and/or immune-mediated disease. Defining the mechanisms driving infection tolerance and persistence within reservoir hosts will reveal novel strategies for viral countermeasures and inform rational surveillance programs. This study was funded by the NIAID/NIH 1R01AI171289-01 grant. No animal studies were performed.

### 2.6. Understanding Viral Immunology and Vaccines

William B. Foreman, Ashley L. Taylor, and Tyler N. Starr of the Department of Biochemistry, University of Utah, performed a study on SARS-CoV-2 monoclonal antibody therapeutics, which have been escaped by viral evolution and rendered ineffective. When they surveyed antibody activity across SARS-CoV-2 and related sarbecovirus lineages, they found that an antibody either binds broadly to multiple variants or potently neutralizes viral entry, but rarely does an antibody exhibit both breadth and ultrapotent neutralization. They investigated a set of promising epitopes within the SARS-CoV-2 receptor binding domain that are functionally constrained and therefore cannot evolve to escape antibody binding with the goal of engineering robust monoclonal antibodies that potently neutralize not only SARS-CoV-2 and its variants but also the broader sarbecovirus lineage. The goal was to use a combination of yeast surface display and deep mutational scanning methods to survey the mutational space of the variable regions of promising parental antibodies. They explored two distinct evolutionary tracks to engineer antibodies that both bind broadly and potently neutralize the sarbecovirus lineage. First, they engineered potent antibodies to bind more broadly, and second, they engineered broad antibodies to more potently neutralize viral entry. They presented initial deep mutational scanning data reporting the impacts of single amino acid mutations across the antibody variable domains and highlighted future plans to express and characterize the final affinity-matured antibodies for breadth, escapability, and neutralization. The proposed work will investigate methods to engineer antiviral antibodies to have both breadth and potency, leading to the development of robust anti-SARS-CoV-2 antibody therapeutics with efficacy against future sarbecovirus spillovers. This work will also inform pan-sarbecovirus vaccine design targets by revealing the limitations for the breadth and potency of these classes of antibodies at these epitopes. No animals or humans were subjects of research in this study. Funding was derived from NIH/NIAID DP2 AI177890 grants.

## Data Availability

No new data were created or analyzed in this study. Data sharing is not applicable to this article.

